# A novel sustainable platform for scaled manufacturing of double-stranded RNA biopesticides

**DOI:** 10.1186/s40643-022-00596-2

**Published:** 2022-10-06

**Authors:** Alison Obinna Nwokeoji, Eleojo Ahuva Nwokeoji, Tachung Chou, Abou Togola

**Affiliations:** 1 Chemical and Biological Engineering, University of Sheffield, Sheffield, S1 3JD UK; 2 School of Biosciences, University of Sheffield, Sheffield, S10 2TN UK; 3 All First Technologies Co. Ltd, No.208, Longnan Rd, Pingzhen Dist, Taoyuan City, Taiwan; 4 International Institute of Tropical Agriculture (IITA) Kano Station, PMB 3112, Sabo Bakin Zuwo road, Kano, Kano State, Nigeria

**Keywords:** RNAi, dsRNA bioprocess, Pest control, Biopesticides, dsRNA purification, Autoinduction media

## Abstract

RNA interference (RNAi) represents one of the most conserved pathways evolved by eukaryotic cells for regulating gene expression. RNAi utilises non-translatable double-stranded RNA (dsRNA) molecules to sequester or degrade mRNA molecules gene. In RNAi, specifically designed exogenous dsRNA delivered to the cell can silence a target gene, a phenomenon that has been exploited in many functional studies and explored in biopesticide applications. The search for safe and sustainable crop pest management options drives the need to offset the effect of inorganic pesticides on biodiversity. The prospect of replacing inorganic pesticides with dsRNA crop spray is gaining popularity, enhanced by its high-target specificity and low environmental impact. However, for dsRNA to reach the pesticide market, it must be produced cost-effectively and sustainably. In this paper, we develop a high-yield expression media that generates up to 15-fold dsRNA yield compared to existing expression media utilising 1 mM IPTG. We also optimise a low-cost purification method that generates high-quality and purified dsRNA. The developed method circumvents the need for hazardous chemical reagents often found in commercial kits or commercial nucleases to eliminate contaminating DNA or single-stranded RNA (ssRNA) species. We also demonstrate that the production platform is scalable, generating 6.29 mg dsRNA from 259 mg wet *E. coli* cell pellet. The results also provide structural insights into the heterogeneous dsRNA species within the microbial-derived dsRNA pool. Finally, we also show that the purified ‘naked’ dsRNA, without prior formulation, can induce insect toxicity under field conditions. This study provides a novel, complete, low-cost process dsRNA platform with potential for application in industrial dsRNA production.

**Graphical Abstract:**

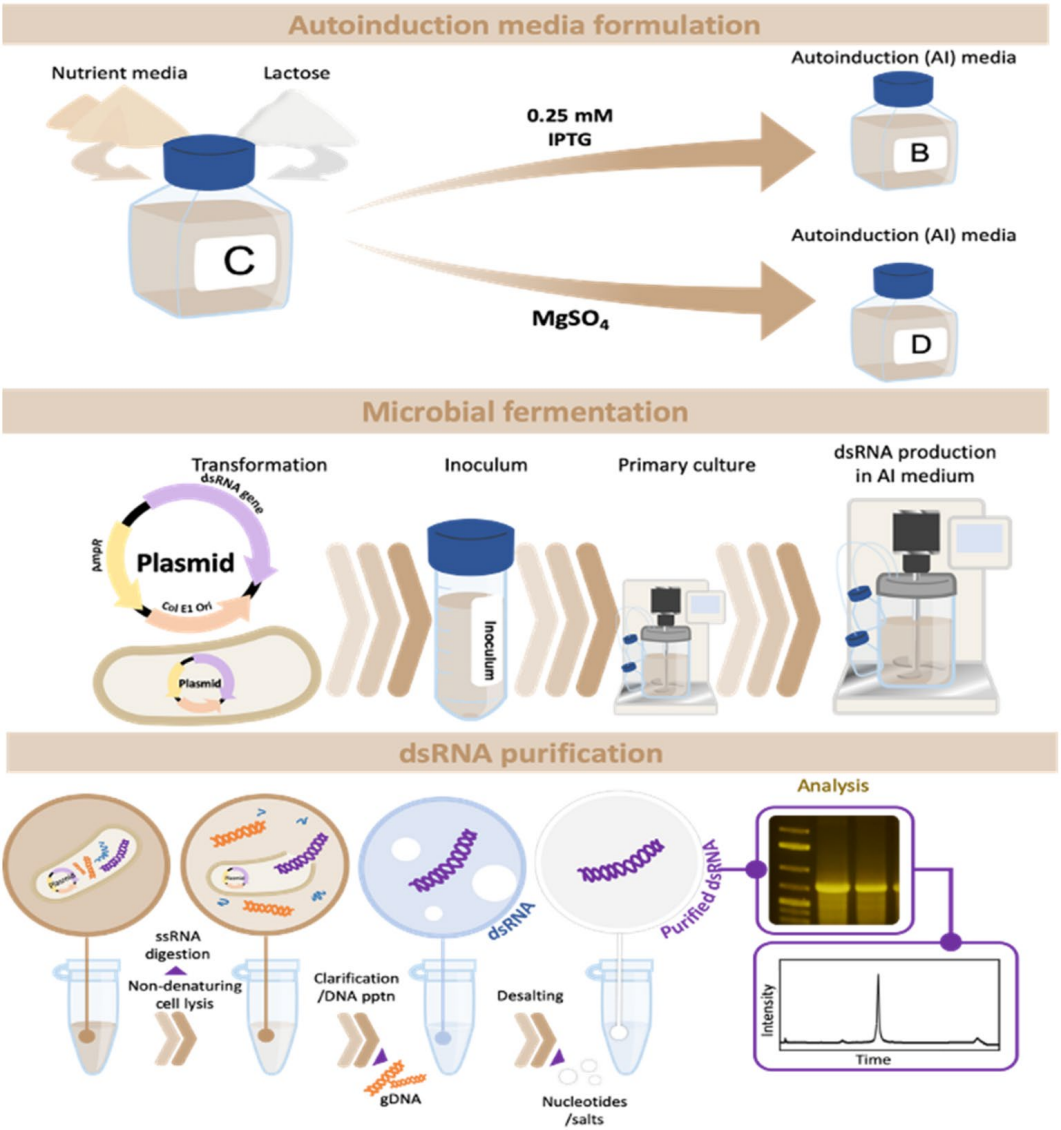

**Supplementary Information:**

The online version contains supplementary material available at 10.1186/s40643-022-00596-2.

## Introduction

RNA interference (RNAi) represents one of the most sophisticated and conserved pathways evolved by eukaryotic cells for controlling gene expression. Although first described in the model organism *Caenorhabditis elegans* (Fire et al. 1998), RNAi is widely found in insects, plants (Gordon & Waterhouse, 2007) and animals (Schuster et al. 2019). In a post-transcriptional gene repression process, RNAi utilises non-translatable double-stranded RNA (dsRNA) molecules to sequester or degrade mRNA molecules. In the RNAi pathway, Dicer, an enzyme member of the RNase III family, cleaves long molecules of dsRNA into short RNA segments (approximately 20 bp long) to form siRNAs. One strand of the siRNA (guide strand) is selectively bound to Argonaute protein (an RNase H-like protein) in the RNA-induced silencing complex (RISC). It guides the complex to the sequence-specific sites resulting in mRNA cleavage in a complementary manner (Zamore & Haley, 2005).

Pesticide toxicity on non-target organisms and environmental pollution are the most urgent concerns in current agriculture. As a result, RNAi technology, with its unprecedented pest target specificity and low environmental impact, is rapidly evolving as a promising safe alternative to toxic inorganic pesticides (Rank & Koch, 2021). Crop spray consisting of “naked”, complexed, or encapsulated dsRNA is becoming the most popular field application for RNAi.

The ability of RNAi pathways to inhibit gene expression holds considerable economic value in crop protection and biological control. Ingestion of exogenous dsRNA induces RNAi-mediated gene silencing in *C. elegans* and many pests (Baum et al., 2007; Leelesh & Rieske, 2020; Timmons et al. 2001). The efficacy of dsRNA-based induced gene silencing in insect pests depends on an efficient administration. Oral delivery of formulated dsRNA and dsRNA-expressing bacteria to larvae effectively causes RNAi-induced mortality in stink bugs (*Euschistus heros*) and emerald ash borer (EAB), respectively (Castellanos et al. 2019). Micro-injection has also been reported (Leelesh & Rieske, 2020). However, efficient dsRNA delivery could significantly depend on the target insect species and tissue. Insect orders respond differently to RNAi (Christiaens et al. 2020; Cooper et al. 2019). Lepidopterans are less sensitive to RNAi (Terenius et al. 2011) partially due to a repertoire of nucleases that degrade and, therefore, deplete dsRNA available to RNAi machinery and processing (Guan et al. 2018; Singh et al. 2017). Increasing dsRNA dosage by several micrograms may be required to achieve significant RNAi silencing in these species in laboratory settings, while field settings may even be more demanding (Xu et al. 2016).

Therefore, the cost of producing RNA represents a significant challenge to deploying dsRNA spray technology. Current methods for dsRNA production include in vitro transcription, chemical synthesis, and microbial fermentation. Recent reports suggest that about 2–10 g of dsRNA may be needed per hectare (Zotti et al. 2018). Chemical synthetic and in vitro transcription approaches can achieve industrial-scale dsRNA production due to their relative ease. However, the prohibitive costs associated with these methods are unsustainable, especially for agricultural applications. The advantages of chemical synthesis are the large yield of high-purity siRNA and a broader range of modifications available than other methods (Amarzguioui et al. 2005; Tenllado et al. 2003). Drawbacks include the price and turnaround times (typically 4–12 days, depending on synthesis and purification options). In vitro transcription (IVT) kit is less expensive than its chemical synthesis counterpart, with the commercial MEGAscript^™^ allowing the production of 1 g at the cost of $3000 within hours. Although IVT is efficient, production costs are unsustainable, especially for pest control applications. Microbial production promises to be less expensive, with target costs projected to be near $4 per gram. To achieve this and make the price commercially feasible, innovations in the microbial dsRNA process, including production and purification procedures, are needed to maximise dsRNA yield and quality.

One microbial system is the bacterial HT115 (DE3) (Takiff et al. 1989) strain of *E. coli*, an RNase III-deficient bacterium. RNase III degrades dsRNA in *E. coli,* and its absence allows dsRNA to accumulate in the cytoplasm. This strain is a derivative of the BL21 DE3 and, as such, expresses the bacteriophage T7 polymerase gene from an inducible (Lac) promoter. Therefore, dsRNA can be “overexpressed” in HT115 strain similarly to microbial recombinant protein overexpression when transformed with expression plasmids containing T7 promoter sequences. The ability to perform large-scale fermentation for dsRNA production raises the possibility of directly feeding dsRNA-expressing bacteria to insects or spraying induced bacteria on crops for pest control applications. (Goodfellow et al. 2019; Kunte et al. 2020; Timmons et al. 2001). Recent studies in lepidopteran pest genera *Spodoptera* and *Helicoverpa* demonstrate the efficacy of this approach (Vatanparast et al. 2021; Wan et al. 2021; Wang et al. 2021).

The application of dsRNA insecticides via direct spraying of the microbial fermentation material (expressing the dsRNA) has potential drawbacks related to crop spraying itself as well as challenges and costs of the formulation. Although this effectively triggers knockdown of some insects' target genes including, haemocoel of orthopteran insects (Verdonckt & Vanden Broeck, 2022), it is less effective in insects such as *Helicoverpa* pests due to immune responses to the presence of bacteria in the haemolymph (Li et al. 2019).

An alternative approach is to extract (and purify) the dsRNA from the fermentation material before formulation and delivery of the dsRNA to the crop. This option is desirable for functional research and requires the administration of pure or “naked” dsRNA via injection or transfection to the research organism. The potential benefits include improved RNAi activity of the active ingredient, reduced formulation cost, enhanced active ingredient delivery, and improved characterisation and quantification of the active ingredient. Administering dsRNA extracted from induced HT115 DE3 induces target gene knockdowns in a lepidopteran species (Wan et al. 2021). However, microbial production of dsRNA growth and expression media may contribute to the cost of production.

Current expression media for dsRNA production in bacteria utilises an artificial lac-inducer, Isopropyl ß-D-1-thiogalactopyranoside (IPTG) (Ahn et al. 2019; Kim et al. 2015). IPTG is highly stable due to low cellular utilisation; however, it is potentially toxic and considerably expensive, especially in the large-scale recombinant protein production (Larentis et al. 2014; Pan et al. 2008). Alternatives to IPTG include lactose which converts allolactose upon cellular uptake and isomerisation by β-galactosidase (Muzika et al. 2018; Wheatley et al. 2013). Lactose uptake in growth media inhibits glucose and repress protein expression (Winkler & Wilson, 1967). Therefore, autoinduction media formulation supports microbial biomass generation and induction. Although commercial autoinduction media are generally cheaper than IPTG, they may still be expensive for large-scale production. With some vendors selling autoinduction media for £530 per Kg, it costs considerably higher than LB media which is sold at £40.36 per Kg by some vendors. Hence, developing inexpensive alternative autoinduction media will drastically reduce the cost of microbial dsRNA or recombinant protein production.

Another significant contributor to the cost of microbial dsRNA production is downstream processes, specifically, dsRNA purification. Existing methods for dsRNA extraction include commercial kits that utilise procedures of varying complexity to extract total nucleic acids (Chomczynski & Sacchi, 1987; Posiri et al. 2013). Therefore, the extracted dsRNA still contains ssRNA and DNA. Moreover, most of these methods use chemicals of significant hazards, such as phenol, chloroform, formamide, and Guanidium Hydrochloride. An inexpensive method that uses less hazardous reagents termed RNASwift was recently developed and demonstrated to be efficient in extracting dsRNA and total RNA (Nwokeoji et al. 2017). It is also possible to use commercial RNA extraction kits with ssRNA-specific ribonucleases and DNases to purify dsRNA (Nwokeoji et al. 2017). However, these commercial ribonucleases will contribute additional costs to the production processes.

In this current study, we developed and tested three different media formulated from lactose and LB for the expression of dsRNA. We show that these media produced up to a 15-fold yield increase compared to an IPTG-based inducible system. These methods can significantly reduce the cost of dsRNA associated with traditional IPTG while substantially increasing dsRNA yield. We also developed a novel method for dsRNA purification that utilises inexpensive, less hazardous reagents (low NaCl, SDS) and does not require commercial nucleases. First, we exploit the chemical lability and susceptibility to *E. coli* ribonucleases to eliminate ssRNA species. The dsRNA is generally more stable and unaffected by these factors. Secondly, selective condensation of higher-molecular weights DNA in high salt and differential solubility of DNA and RNA at low pH enables precipitation of DNA on silica matrix and removal. We show this optimised procedure is high yield and produces high-quality and -purity dsRNA.

## Materials and methods

### Chemicals and reagents

Ampicillin sodium salt, tetracycline hydrochloride, Isopropyl β-D-1-thiogalactopyranoside (IPTG) ≥ 99%, LB Broth Miller (Formedium), sodium dodecyl sulphate (SDS), 99%, sodium chloride (NaCl), 99% were all obtained from (Sigma-Aldrich, Poole, UK). Nucleic acids were analysed by IP-RP-HPLC on an Agilent 1100 series HPLC using a Proswift RP-1S Monolith column (50 mm × 4.6 mm I.D. ThermoFisher) and buffers prepared with (TEAA) pH 7.0 (Fluka, UK), acetonitrile (ThermoFisher), and HPLC grade water for nucleic acid analyses. Reagents used for purification and mass mapping of dsRNA RNase T1 (100 U/uL, Ambion), RNase A (1 mg/mL, Ambion), and DNase I (Ambion). Oligonucleotides were analysed on the U3000 HPLC system (Thermo Scientific) and maXis Ultra High-Resolution Time of Flight Instrument (Bruker Daltonics) using the Accucore C18 column (150 mm × 2.1 mmID).

### Gene sequence retrieval and construction of plasmid-bearing dsRNA gene sequence

The sequences of *Bemisia tabaci* acetylcholinesterase ecdysone receptor (EcR), ribosomal protein L9, ecdysone receptor-like (LOC109036570), and V-ATPase identified with the BLAST (Altschul et al. 1990) algorithm using orthologous genes from closely related species or model organisms as queries and searching against transcriptome databases. Partial CDS of these genes were selected and manually verified for open reading frames (ORF) and protein translation using Snapgene (Insightful Science). These partial sequences were linked to create a *B. tabaci* multigene-targeting 706 bp known as AREV4. Another dsRNA gene sequence, ATU1, was constructed by selecting the complete CDS of *Bemisia tabaci* Alpha-tubulin. T7 RNA promoters and synthetic terminators flank both ends of the synthetic dsRNA genes. These were then synthesised by GeneArt^®^ Gene Synthesis (Invitrogen Life Technologies) and cloned into plasmid, pMA-X series supplied by the vendor.

### Induction and autoinduction media formulation

A base autoinduction media (AI) is formulated by mixing modified Luria Broth (LB) media (25 g/L) and lactose (6.25 g/L). The following five media were prepared and tested for their efficacy in dsRNA expression: media A (contains 1 mM IPTG and modified LB), media B (contains 0.25 mM IPTG and base autoinduction media), media C (base autoinduction media), and media D (base autoinduction media and MgSO_4_) and E (contains 0.25 mM IPTG and modified LB).

### Expression of dsRNA gene using *E. coli* HT115(DE3)

The *E. coli* strain, HT115(DE3) (Timmons et al. 2001) obtained from Cold Spring Harbor Laboratory, NY, USA, was transformed with pAREV4 and pATU1. A 5 mL LB media containing 10 ng/mL tetracycline and 100 µg/mL ampicillin was inoculated with a colony from the transformed cells and incubated overnight at 37 °C at 200 rpm. 50 mL LB media containing the same antibiotic concentration was seeded with the overnight culture, incubated at 37 °C, and allowed to reach an OD600 nm of 0.8. Cells are induced by adding an appropriate volume of 10X autoinduction media to the culture and incubating at 37 °C for 3 h, 6 h, and 18 h as experimentally required.

### Ion-pair reverse-phase high-performance liquid chromatography (IP-RP-HPLC)

IP-RP-HPLC was performed on a passivated Agilent 1100 series HPLC for all samples using a Proswift RP-1S Monolith column (50 mm × 4.6 mm I.D. ThermoFisher). The HPLC analysis generates chromatograms at UV 260 nm under 50 °C column temperature using the following buffer conditions: Buffer A contains 0.1 M triethylammonium acetate (TEAA) pH 7.0 (Fluka, UK), while Buffer B has 0.1 M TEAA, pH 7.0, and 25% acetonitrile (ThermoFisher). Gradients used for RNA separation and analysis are as follows: gradient started at 22% buffer B to 27% in 2 min, followed by a linear extension to 62% buffer B over 15 min, then to 73% buffer B over 2.5 min at a flow rate of 1.0 ml/min at either 50 °C.

### Total RNA and dsRNA extraction

Induced *E. coli* cells were harvested by centrifugation at 7500 rpm at 20 °C for 5 min. Total RNA extraction was performed using a previously described RNA extraction method, RNASwift (Timmons et al. 2001). Following RNA extraction, samples were treated with RNase T1 (Ambion) and DNase 1 (Ambion) before solid-phase extraction using a silica spin column, as described previously (Nwokeoji et al. 2016).

### Development of low-resource dsRNA purification method (DPM1)

1 mL culture of *E. coli* cell samples (OD 0.5–1.18) was harvested by centrifugation at 7500 rpm at 20 °C for 5 min. The harvested *E. coli* cell pellets were suspended in 100 µl 5 mM EDTA (pH 8.0) and incubated for 10 min (L1). 50 μL of solution A (containing 25 mM NH4(SO4)2) was added before incubation at room temperature for 20 min (CL1). 50 μL 2% SDS was added and incubated for another 20 min (L2). 200 μL 5 M NaCl was added, mixed by inverting tube 10 times, and centrifuged for 10 min at 10,000 pm. To filter off residual cell debris and remove DNA, the cleared supernatant into the new tube and the clarified supernatant mixed with 100 μL pre-washed silica media and centrifuged for 1 min. Approximately 400 μL of the supernatant was collected, combined with 200 μL isopropanol, and centrifuged at 13,000 rpm for 20 min. Discard supernatant and centrifuge again to remove residual isopropanol. The recovered RNA pellet was dissolved in 150 µL water.

### Optimised low-resource dsRNA purification method (DPM2)

The optimised purification method retains the lysis method established in DPM1. However, in addition to the L1 step, 100 μL solution A2 (containing 25 mM NH4(SO4)2 and 1% SDS) was also tested, which combined CL1 and L2 (L1-2). CL1 and L2 or CL1-2 steps were followed by adding 200 μL 5 M NaCl at pH 6, 5.5, and 5.0. The NaCl solution pH was adjusted using HCl (Sigma). Subsequent steps in the procedure are the same as described in DPM1 and purified dsRNA pellet dissolved in 150 μL HPLC grade water (ThermoFisher Scientific).

### Scaled dsRNA purification (sDPM1 and sDPM2)

100 mL culture of *E. coli* cells was harvested by centrifugation at 7500 rpm at 4 °C for 5 min. Cell pellet harvested from culture and induced with media C (OD 1.18) was suspended in 10 mL mM EDTA (pH 8.0) and incubated for 10 min (L1 step). For sDPM1, 5 ml solution A (containing 25 mM NH4(SO4)2) was added before incubation at room temperature for 20 min. 5 mL 2% SDS was added and incubated for another 20 min (L2) before adding 20 mL 5 M NaCl and centrifuged for 20 min.

For sDPM2, 10 mL solution A2 (containing 25 mM NH4(SO4)2 and 1% SDS) was added and the mixture incubated for 20 min prior to addition of 20 mL 5 M NaCl (pH 5.5) and centrifugation at 20 min.

Subsequently, supernatants recovered from both methods were mixed with pre-washed 1 mL silica and centrifuged. Approximately, 35 mL supernatants were recovered from samples in both procedures, combined with 15 mL isopropanol, and centrifuged at 13,000 rpm for 20 min. The supernatant was discarded, and the tube was centrifuged again to remove residual isopropanol. The RNA pellet was dissolved with 8 mL HPLC grade water (ThermoFisher Scientific).

### Analysis of RNA quality and quantity

The quality and quantity of RNA were determined using a NanoDrop^™^ 2000c spectrophotometer (Thermo Scientific). RNA concentrations were determined from the absorbance at 260 nm. The A260/280 nm and A260/230 nm ratios were obtained using the NanoDrop™ instrument. RNA quality was determined by performing ion-pair reverse-phase chromatography using a 10 µl injection from the eluted/resuspended RNA. In experiments involving replicate samples (quadruplicate), an equal volume of dsRNA extracted from replicates was pooled, and 10 μL of this was injected into the HPLC. The relative amount of intact dsRNA was measured by determining the dsRNA peak area derived from the IP-RP-HPLC trace. Subsequently, dsRNA percentages were determined using the chromatographic RNA peak areas.

### Efficacy test for dsRNA

A toxicity test was carried out at IITA, Nigeria, to determine the efficacy of the dsRNA as insecticides against *Bemisia tabaci*. The dsRNA samples used were purified using the DPM1 method described in materials and method. Ten fast-moving insects (used as an indicator of good health) were selected randomly, captured using an aspirator and introduced in a vial containing cowpea leaves covered with the dsRNA solution. The experiments were performed in four replicates with ten (10) insects per replicate and different concentrations of the purified dsRNA tested. Distilled water was used as a check (negative control). Mortality was observed over three days, and live insects were counted each day starting from the second day.

### Statistical analysis

Statistical analysis was accomplished using Graphpad Prism 9. Two-way analysis of variance (ANOVA) and two independent sample t-tests were performed to quantify the considerable differences between different conditions. Pearson correlation analysis was performed to evaluate the accuracy and consistency of the quantification methods. All experiments were conducted in either triplicates or quadruplicates to obtain statistically significant results.

## Results

### Simple and cost-effective autoinduction media for dsRNA production

**Fig. 1:**
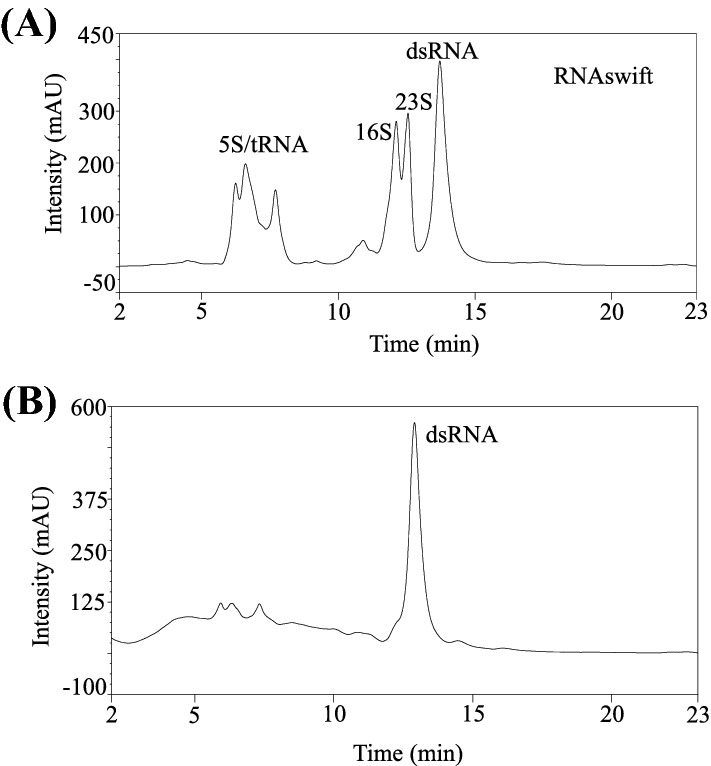
IP-RP-HPLC Analysis of dsRNA. **A** Chromatogram of total RNA extracted from HT115 DE3 expressing AREV4 dsRNA (induced with media C) using RNASwift extraction method **B** Chromatogram of dsRNA purified from HT115 DE3 expressing AREV4 dsRNA (induced with media C) using RNASwift in conjunction with commercial ribonucleases

We formulated an autoinduction media by incorporating lactose into modified LB expression media to develop an inexpensive expression system for dsRNA. Following primary and secondary culture to generate sufficient biomass of the *E. coli* strain harbouring plasmid for dsRNA, cells were induced in AI media for 12 h to express dsRNA. Total RNA was extracted from cells using RNAswift, and IP-RP analysis shows a peak corresponding to the dsRNA and peaks for the rRNAs (Fig. 1a). The peak corresponding to the dsRNA has a retention time of about 14 min on the gradient we used.

We treated the extracted RNA with RNase T1 before IP-RP HPLC analysis to confirm that the observed peak is the dsRNA target. The hypothesis is that as RNase T1 is demonstrably specific for ssRNA if the observed peak is ssRNA species, it will degrade; otherwise, it will be intact. In this experimental setup, the RNase T1 cleaves ssRNA species, including single-stranded regions of rRNAs, providing an internal control for the RNase T1 enzyme activity. The result demonstrates that the observed peak is dsRNA (Fig. 1b).

These results demonstrate that the AI media effectively induces the lac operon and suggest that this system may be a valuable alternative to IPTG-based induction. Therefore, one of this study’s main objectives is to test the yield and quality performance of different formulations of these expression media. However, an adequate dsRNA purification method is essential to effectively perform these analyses and ensure that all processes involved in the platform are sustainable.

### Development of a cost-effective, less hazardous dsRNA purification method

To accurately quantify and assess the quality of dsRNA produced in our formulated media, it was necessary to utilise a dsRNA extraction method to remove contaminants, ssRNA, and DNA effectively. Existing methods for dsRNA purifications use expensive commercial DNases and RNases to eliminate ssRNA and DNA, respectively. Moreover, commercial extraction methods rely heavily on organic solvents with safety, environmental, and cost implications. For low-cost production of dsRNA, reduced use or elimination of these factors is essential. Therefore, developing a robust, high-yield, commercial nuclease-free, and low-organic solvent method will enable purification and accurate dsRNA quantification while reducing production cost—an essential objective in this study.

### Novel dsRNA purification platform free from commercial nucleases and organic solvent (DPM1)

To develop a commercial-nuclease-free method for dsRNA isolation, we exploited the substantially higher resistance of dsRNA to bacterial ribonucleases when compared to ssRNA. We hypothesised that first incubating bacterial cells in 10 mM EDTA would result in cell lysis, ribonuclease release, and selective degradation of ssRNA. Second, DNA condenses and readily salts out in high-saline, low-pH conditions on silica matrix.

**Fig. 2:**
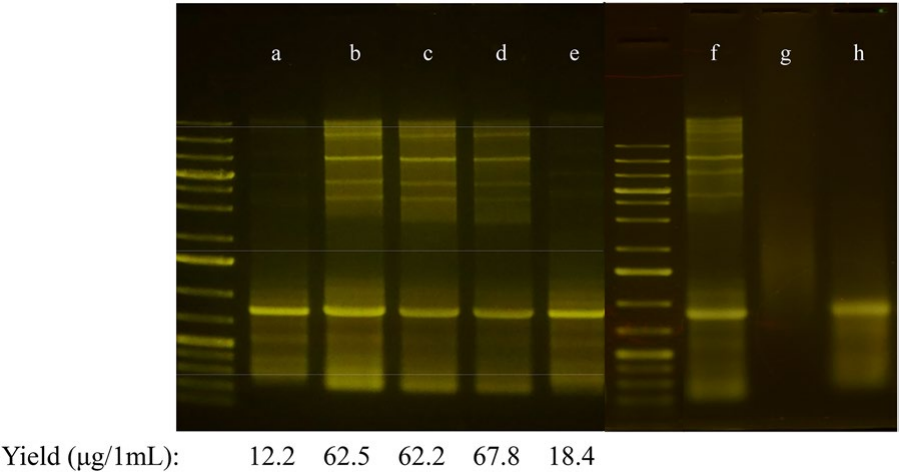
Purification of AREV dsRNA. Method DPM1 was used to extract 700 bp (AREV4) dsRNAs from 1 mL culture induced for 18 h in the media A–E and extracted RNA from the media analysed by agarose gel electrophoresis: A lanes a, b, c, d and e: RNA sample from media A, media B, media C, media D and media E, respectively. For lanes f, g and h, media B purified RNA samples were treated as follows before agarose gel electrophoresis: f) added 1 µL RNase T1 and incubated for 10 min g) mixed with DMSO (50% final concentration), incubated at 90 °C for 1 min, added 1 µL RNase T1 and incubated for 10 min h) mixed with 50% DMSO and incubated at 90 °C for 1 min)

DPM1 described in the method section was used to extract dsRNA from cells grown in five formulated media. Following analysis, the result (Fig. 2A) shows that the method effectively purifies dsRNA irrespective of expression media. Interestingly, agarose gel analysis suggests that the purified dsRNA from media A and E (media containing IPTG-induced lanes a and e) seems more homogenous than those from media B, C, and D (lanes b, c, and d). RNA extracted from E. coli generated in this media has multiple but less intense high-molecular-weight bands in addition to the expected prominent dsRNA band.

RNase T1 cleaves ssRNAs but can also cleave dsRNA when denatured with DMSO in conjunction with heat, as established in (Timmons et al. 2001). RNase cleavage assays were performed to demonstrate that these higher-molecular weight bands are not single-stranded RNA species. A purified dsRNA sample from media B was treated with RNase T1 to test if these multiple bands are ssRNA species. The result shows that the multiple bands remain intact (Fig. 2, lane f), suggesting that these are either dsRNA or DNA bands. Therefore, to determine if these are dsRNA or DNA, the same samples were denatured in DMSO before RNase T1 treatment. If DNA, these bands should remain intact, but if not, there would be no detected bands. The result shows no bands (Fig. 2A, lane g), demonstrating that the higher-molecular weight molecules are indeed dsRNA. Denaturation with DMSO and heat without RNase treatment shows the disappearance of the high-molecular-weight bands, with the “main” band at 700 bp appearing broader and less fluorescence. This result suggests that DMSO denatures these multimeric dsRNA structures causing them to dissociate into monomeric units with the same electrophoretic mobility as the “main” band. The broadness of the 700 bp band is consistent with the notion that increased monomer populations with the same electrophoretic rate. These results are consistent with observed multimers in a previous study (Nwokeoji et al. 2019).

**Fig. 3:**
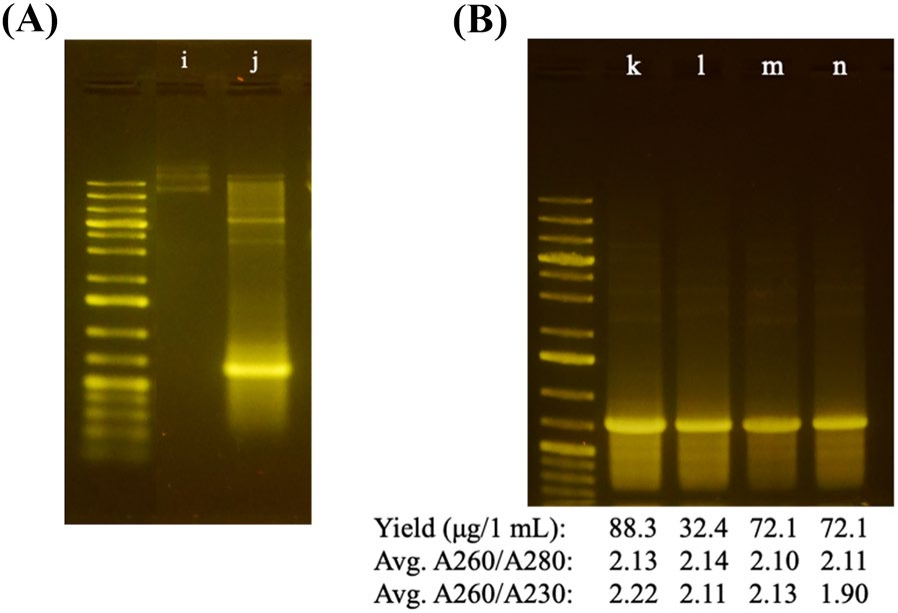
Optimisation of purification method to reduce background multimeric dsRNA (DPM2). Method DPM2 was used to extract 600 bp dsRNA (ATU1) from cells bearing ATU1 construct and induced in media C. **A** i) uninduced cell, j) media C. **B** dsRNA extraction performed using DPM and eluted with 150 uL nuclease-free water with modifications as follows: k) NaCl solution adjusted to pH 6.0 (588.6 ng/uL, l) NaCl solution adjusted to pH 5.5 (215.8 ng/uL) m) NaCl solution adjusted to pH 5.5 (NH4)SO4 and SDS step performed together resulting in more prolonged incubation of sample in both, 480.3 ng/uL. n) NaCl solution adjusted to pH 5.0

The efficiency of the developed method to purify dsRNA was further tested by “extracting” RNA from uninduced cells. The uninduced cells express no dsRNA, and extraction will yield no nucleic acids if the method efficiently removes non-specific nucleic acids. The result shows no discernible band on the agarose gel apart from a few faint bands corresponding to *E. coli *genomic DNA (Fig. 3A, lane i) in contrast with the induced cells (Fig. 3A, lane j). The result demonstrates that the procedure effectively removes single-stranded RNA and DNA. Observed genomic DNA band in uninduced cells suggests retention of residual genomic DNA in the absence of dsRNA. The multimeric structures in both dsRNA samples, AREV4 and ATU1, indicate that most dsRNA sequences may form these structures but in differing amounts.

### Optimisation of the dsRNA purification method (DPM2)

The observed multimer may include dsRNA–dsRNA and residual RNA–DNA hybrids. The result suggests that if these residual RNA–DNA hybrids contaminate the purified dsRNA samples, they are insignificant as the DNA component is not observed on the gel after RNase T1/DMSO treatment, even with the highly sensitive Midori stain.

To obtain a more homogenous dsRNA population, we further exploit the differential solubility of DNA and RNA at low pH. We hypothesise that the solubility of the dsRNA/DNA complexes reduces with decreasing pH. We modified the DPM1 procedure to test this hypothesis by adjusting the clarification buffer to pH 6—5. At pH 6, the multimeric structures were significantly reduced (Fig. 3A).

As the pH approached 5, all multimeric structures were barely discernible on the gel using the highly sensitive Midori dye. For ease, we term this second method DPM2. Comparing the two methods for dsRNA extractability suggests that DPM2 performs better and produces a larger yield of high-purity dsRNA at pH 6 and 5.5 (Fig. 3A, lane j, and 3B, lanes k, l, and m).

Even though pH 5 yields the most homogenous dsRNA (Fig. 3B, lane n), the optimum yield and purity (determined by evaluating the A260/A230) are at pH 5.5. We performed DPM2 at pH 5.5 in two ways (see Methods section): in one approach, the lysis steps, CL1 and L2, are performed separately, and the other in a single step (CL1–L2). The result shows that the single-step approach produces better yield and purity than the multiple-step lysis. Therefore, we used this high-performing DPM2 method in subsequent experiments to investigate the performance of the various developed autoinduction media for dsRNA expression.

### Optimising a high-yield system for dsRNA production

This study aimed to develop an inexpensive but high-yield alternative expression system. Since the formulated expression system showed an excellent yield, performing a comparative quantitative analysis of dsRNA yield from different media was necessary. The strategy is first to determine the overall impact of IPTG induction on dsRNA at other different induction points and then assess the effect of media composed of only formulated autoinduction media or this in conjunction with IPTG or MgSO_4_.

### Effect of IPTG on dsRNA yield

**Fig. 4:**
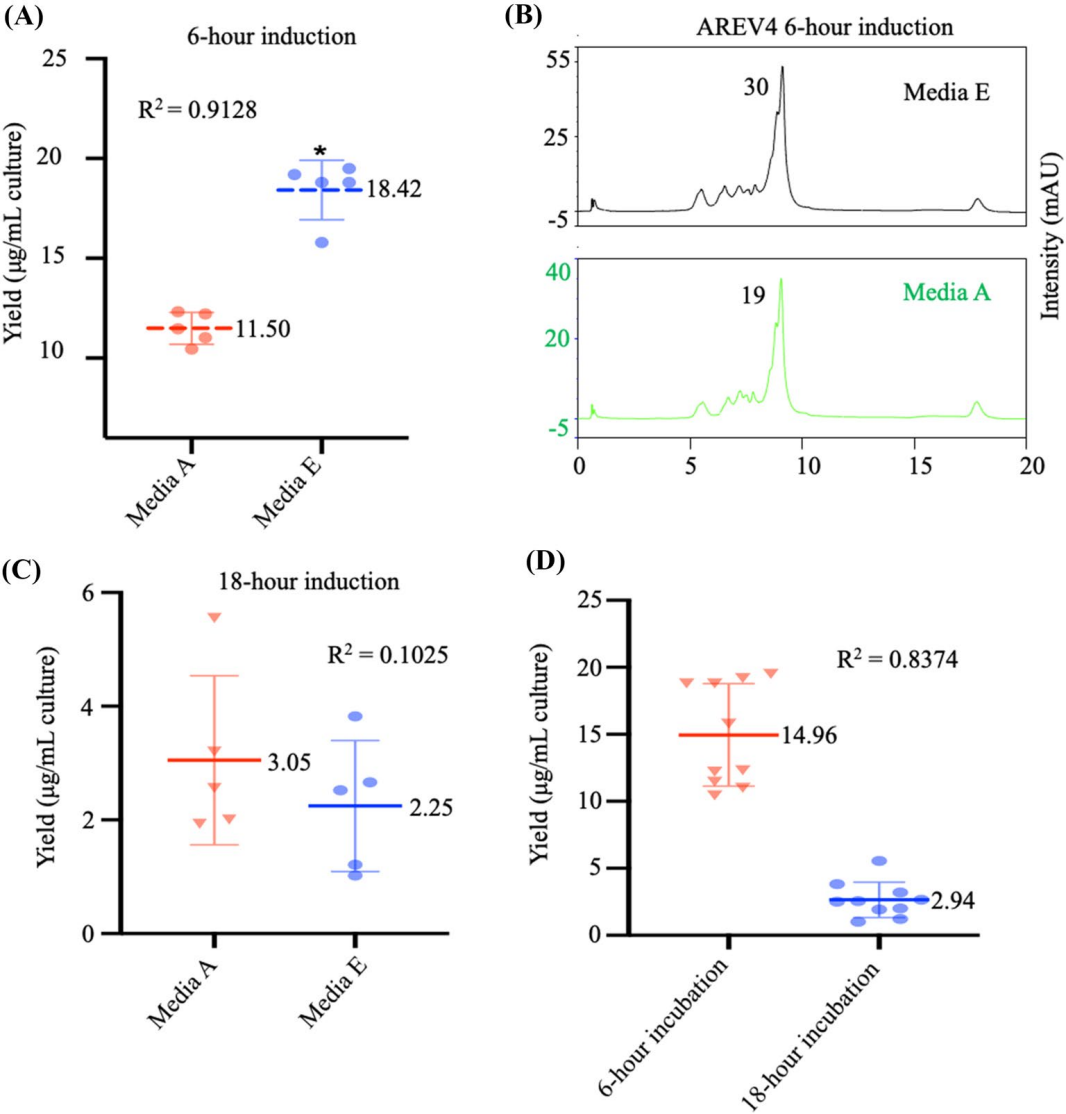
Effect of IPTG and induction time on dsRNA yield. AREV4 dsRNA construct was used in this experiment. Samples prepared in five replicates **A** *t*-test analysis comparing AREV4 dsRNA yield produced from 6-h induction in media A and E suggests no significance between the two media (*t*(8) = 9.149, *p* = 0.2490) with mean values for A and E = 11.50 and 18.42, respectively. **B** The relative yield of purified AREV4 dsRNA produced in media A and E expression systems was determined by measuring the main peak area (value of area indicated) chromatogram obtained by IP-RP-HPLC analysis. **C** t-test analysis of AREV4 dsRNA yield produced from 18-h induction in media A and E suggests no significance (*t (8)* = 0.9559, *p* = 0.6317) between the two media with mean values for A and E = 3.05 and 2.25, respectively. **D** t-test analysis of AREV4 dsRNA yield comparing 6-h induction vs 18-h induction using combined replicate samples (from media A and E) suggests there is a significant difference between the effects of length of induction times (*t(18)* = *9.630*, *p* = 0.0042) with mean values for 6-induction and 18-h induction = 14.96 and 2.94, respectively

Initially, we tested the performance of LB media culture induced with 0.25 mM IPTG (media E) and culture induced with 1 mM IPTG (media A), which are concentrations of IPTG popularly used for recombinant *E. coli* protein overexpression in the literature. Here, we expressed a 680 bp dsRNA sequence (AREV4) in five media E and A replicates. After 6-h induction, analysis shows an average dsRNA yield of 18.42 μg and 11.50 μg for 0.25 mM and 1 mM IPTG induction, respectively (Fig. 4A, Additional file 1: Table S1). The t-test analysis confirms that culture induced with 0.25 mM IPTG for 6 h produced significantly more dsRNA than 1 mM IPTG-induced culture by a difference of 6.924 ± 0.7568 μg (Fig. 4A).

To further validate this, we analysed dsRNA produced from the 6-h induced A or E media by injecting 10 uL of each sample and measuring the area of the prominent dsRNA peak. The result shows that the media E sample (29.19) has a greater dsRNA peak area than media A (Fig. 4B). Taken together, these result shows that 0.25 mM IPTG produces a better yield of dsRNA than 1 mM IPTG.

To determine the impact of elongated IPTG induction on dsRNA, we quantified dsRNA purified from 18-h IPTG-induced cells. The average dsRNA yields obtained are 2.25 μg and 3.05 μg for media E (0.25 mM IPTG) and media A (1 mM IPTG), respectively (Fig. 4C, Additional file 1: Table S1). The t-test analysis reveals no significant difference between the dsRNA yields of media E and A after 18-h induction. Interestingly, these mean values (2.25 and 3.05) are significantly lower than the yield obtained for 6-h induction (18.42 and 11.50). T-test analysis of the 6-h and 18-h induction also shows a significant yield difference between these two periods with the former producing a higher dsRNA yield (Fig. 4D). The low yield obtained after 21-h induction was unexpected. Our initial assumption was that the worst outcome from prolonged IPTG induction would be saturation of *E. coli* with dsRNA inhibiting cell growth. The dsRNA loss over the 18-h IPTG induction period was unexpected, and it was not initially clear why. However, we speculated that either the dsRNA sequence (AREVA) used in this experiment or the IPTG may be toxic to cells when accumulated and causes cell death/lysis, which in turn will result in losing accumulated dsRNA to the culture media.

### Formulated autoinduction media increases total dsRNA yield

**Fig. 5:**
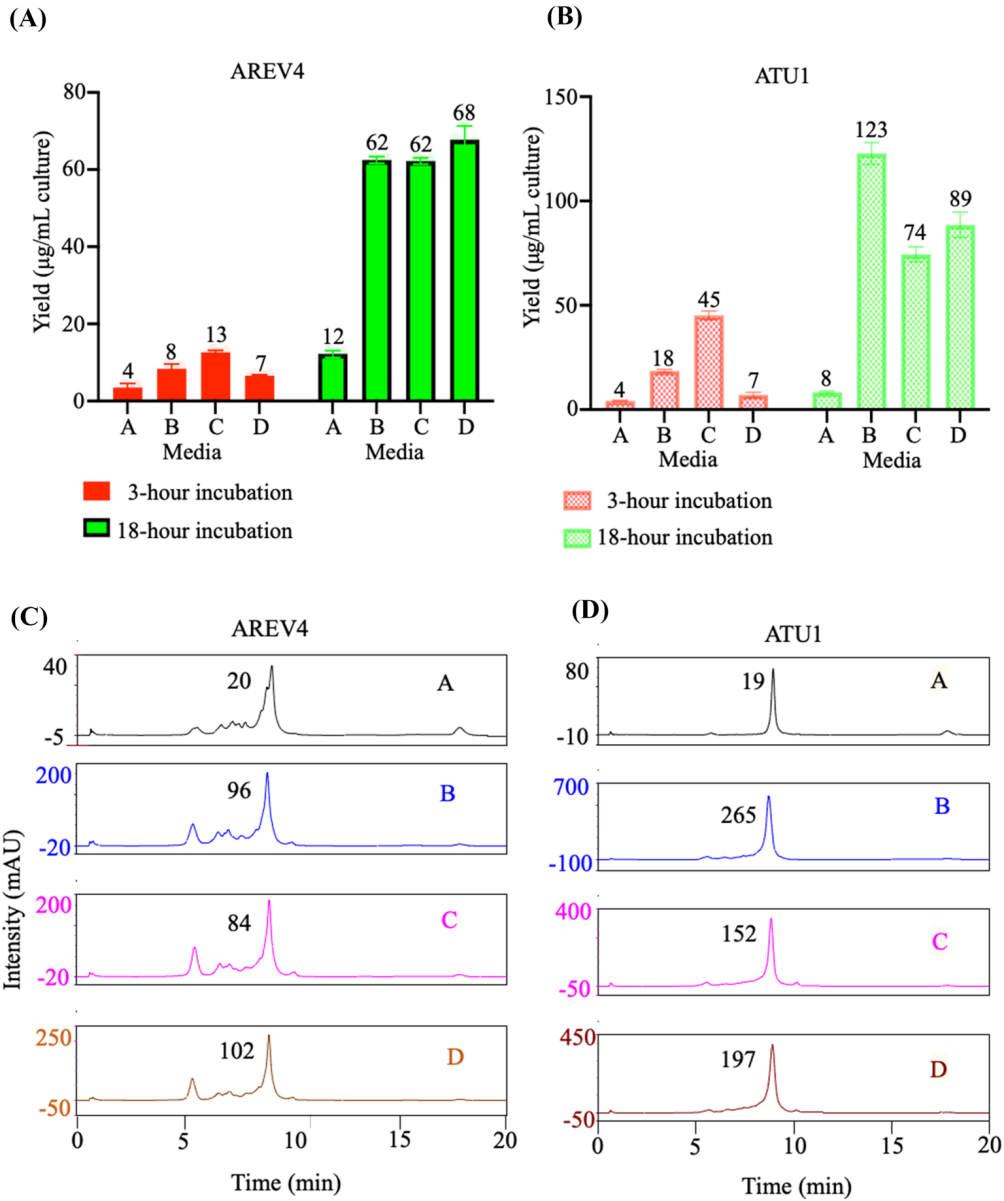
Comparative analysis of dsRNA yield obtained from formulated autoinduction media. **A**, **B** Separated bar graph comparing dsRNA yield (sequences termed AREV4 and ATU1, respectively) purified from cells induced in media **A**–**D** for 3- and 18 h (the bar graphs are labelled with the mean dsRNA yield (μg per mL cell culture), **C** & **D**) AREV4 and ATU1 dsRNA purified from cells induced in media **A**–**D** for 18 h were analysed by IP-RP-HPLC and the area of the main peak determined (peaks labelled with the values of their areas)

In a quadruplicate experiment, we induced cells expressing AREV4 dsRNA in the four autoinduction media, A, B, C, and D (see materials and methods), for 3 or 18 h. This time, a 3-h instead of 6-h induction was chosen to provide a variety of time-course while also capturing the earliest significant dsRNA titre yield from the expression media. Following expression, dsRNA was purified from 1 mL cell culture and quantified on a nanodrop spectrophotometer. Using ANOVA, analysis reveals that both incubation time and induction media type significantly affect yield (Additional file 1: Table S2, S3, and S4). The 18-h induced media A containing 1 mM IPTG produced significantly more dsRNA (mean titre of yield 12.21 μg/1 mL *E. coli* culture) than 3-h induced media A (3.50 μg/1 mL *E. coli* culture) but lower yield than 6-h induced media E (Fig. 5A; Additional file 1: S2, S3, S4, and S5). Cells induced for 18 h in media B–D produced substantially higher dsRNA yield (62.49–67.79 μg) than 3-h induced cells in the same media (3.50–6.57) (Fig. 5A).

Comparative analysis shows that media A had significantly less dsRNA than B–D at 3-h and 18-h induction points (Fig. 5A; Additional file 1: Table S2, S3, and S4). For the 18-h expression, media B–D (titers of B, C, and D of 62.49, 62.22, and 67.79 μg per mL culture, respectively) produced over fivefold dsRNA increase compared to media (12.21 μg) (Fig. 5A).

From the spectrophotometric analysis at 18-h induction, media D has the highest dsRNA titre while media B perform jointly with media C as second best; there was no significant difference between their means (B has a slightly higher mean).

Similarly, we compared yield at 3-h induction with analysis revealing media C as the most productive and D the least (Additional file 1: Table S3). Interestingly, comparative analysis shows that media D, which had the most significant yield after 18-h expression, performed similarly to media A at 3-h induction (Additional file 1: Table S3). The result suggests that the rate of dsRNA expression in media D at the beginning of induction is initially low but increases substantially over time and supports stable production of dsRNA even after prolonged culture incubation.

In Sect. Chemicals and reagents, we speculated that either accumulation of the dsRNA or IPTG or both factors may have been responsible for cell toxicity, death, and lysis leading to dsRNA loss. The fivefold increase in yield after 18-h induction in lactose-containing media B–D demonstrates that lactose improves dsRNA yield. The higher dsRNA titre for lactose-based media contrasts with the low quantity of dsRNA produced in IPTG systems. These data strongly support the notion that IPTG is responsible for cell toxicity and death after prolonged induction.

### Validation of the result obtained for formulated media using a different dsRNA sequence

To further validate the experimental result and determine the effect of dsRNA sequence on yield, we used a different plasmid construct (pMA-ATU1) containing the dsRNA gene termed ATU1 this time. Media A performed the least at both 3 h and 18 h of induction (Fig. 5B; mean yield 4.05 and 8.33 μg/1 mL culture, Additional file 1: Table S6). Although the 18-h induction appears to produce a higher yield, ANOVA suggests no significant difference between the two means (Fig. 5B, Additional file 1: Tables S6, S7). This result contrasts with AREV4, where the dsRNA yield at 18-h induction is significantly higher than the yield for 3-h induction. However, as the ATU1 yield at 18-h induction is comparatively lower than the corresponding yield of AREV4, the result is consistent with the notion of a sustained dsRNA yield loss over prolonged IPTG induction.

Additionally, the 18-h induction for media B–D is extremely significantly higher than the 3-h induction. At 3-h induction, media C performs highest (45 μg), followed by B (18 μg) and D (7 μg) (Fig. 5B). This trend is consistent with the result obtained for AREV4 (in Fig. 5A).

However, in contrast to AREV4 dsRNA, media B appears to produce a significantly highest yield at 18 h (122.9 μg/2 mL culture), followed by media D (88.60 μg/2 mL culture) and C (74.45 μg/2 mL culture) (Fig. 5B, Additional file 1: Table S6, S7, S8, and S9).

### IP-RP HPLC analysis of intact dsRNA further validates that formulated autoinduction media increases yield irrespective of sequence

As established here, the developed dsRNA purification method eliminates single-stranded RNA species and DNA. However, the purified product is total dsRNA, containing both intact dsRNA and incomplete or degraded dsRNA transcripts. From the result obtained, we have established that formulated autoinduction media produce at least a fivefold increase in total dsRNA yield irrespective of the dsRNA sequence. However, it is unclear from the data if this holds for intact dsRNA fraction in a purified dsRNA population that is a mixture of full-length and incomplete dsRNA transcripts.

Therefore, we analysed purified dsRNA samples by IP-RP-HPLC to quantify dsRNA and assess dsRNA quality. For quadruplicates, we pooled replicate samples (as described in the method section) and injection 10 μL into the HPLC. The chromatogram profile for all samples is consistent with purified dsRNA characterised by the absence of ribosomal RNA species. Typically, there is a “main” dsRNA peak corresponding to intact dsRNA and regions of the chromatogram with smaller peaks corresponding to shorter dsRNA or incomplete dsRNA transcripts. The chromatograms obtained for both AREV4 and ATU1 dsRNAs in conjunction with spectrophotometric analysis of sample purity suggest that dsRNA is pure, containing intact dsRNA peak and incomplete or shorter dsRNA transcripts.

We obtained the relative quantification of the intact dsRNA for both AREV4 and ATU1 samples (18-h induction) by measuring the area of the ''main'' dsRNA peak corresponding to intact dsRNA. For AREV4, media D has the highest dsRNA yield (101.79 (mAU), followed by media B (95.53 mAU) and media C (84.29 mAU), while media A has the lowest yield (19.69 mAU) (Fig. 5C). The result suggests the dsRNA yield for media B–D is almost fivefold higher than media A (1 mM IPTG). For ATU1, media B has the highest yield, followed by media D and C. In contrast, media A has the lowest amount of dsRNA (Fig. 5D).

The IP-RP-HPLC analysis of the peak area shows that the relative yield of intact dsRNA obtained from Media B (265 mAU), D (197 mAU) and C (152 mAU) are approximately 14-fold, ten-fold, and eight-fold higher than the yield from media A (19 mAU). For both AREV 4 and ATU1, media C appears to occupy the third position, whereas media D and B occupy the top position for AREV4 and ATU1, respectively. These results are consistent with data obtained from the spectrophotometric analysis. The strong correlation between spectrophotometric and HPLC data for AREV4 and ATU1 (Additional file 1: Fig. S1 A and B) shows that both methods accurately quantify dsRNA and further validate the significance of the comparatively several-fold dsRNA yield increase obtained for the formulated autoinduction media.

### Scalable dsRNA production and purification platform

We performed scaled dsRNA purification on AREV4-expressing cells derived from 100 mL induced culture (media C) to assess the purification methods' scalability. For scale dsRNA purification, we adjusted reagent volumes used in the small-scale (1 ml culture) purification. For example, since we used 100 μL suspension buffer (10 mM EDTA) for the cell pellet recovered from 1 mL, we then used 10 mL suspension buffer for the 100 mL culture; the procedure for both sDPM1 and sDPM2 remained the same.

**Fig. 6:**
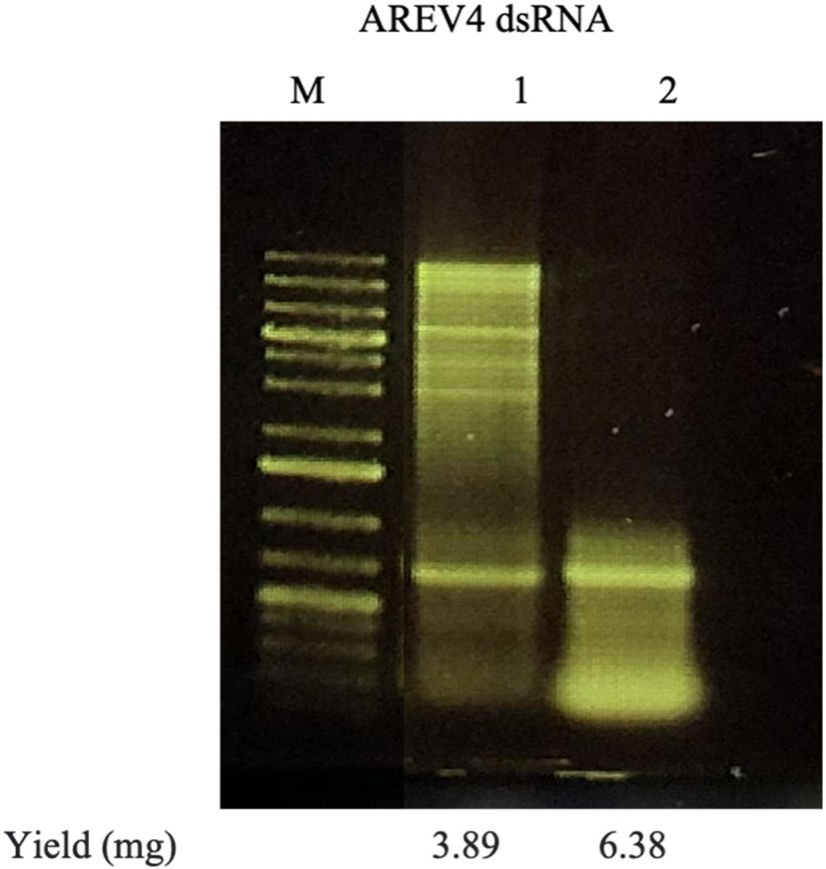
Agarose gel analysis of dsRNA purified using scaled DPM2 purification method. A cell pellet of approximately 259 mg wet weight was generated from media C and dsRNA was purified using either the DPM1 or DPM2 method. Lanes 1 and 2 are dsRNA samples purified using DPM1 and DPM2, respectively

The cell wet weight from the 100 mL media C was approximately 259.60 mg. The yield obtained for sDPM1 and sDPM2 is 3.89 mg and 6.384 mg dsRNA per 259.60 mg wet cell pellet, respectively. The results demonstrate that both sDPM1 and sDPM2 extract dsRNA with sDPM2 producing higher dsRNA titre and lower background dsRNA multimers (Fig. 6). Overall, these show that both purification methods developed in this study are scalable, but sDPM2 of higher efficiency.

### Purified dsRNA impact insect mortality

**Fig. 7:**
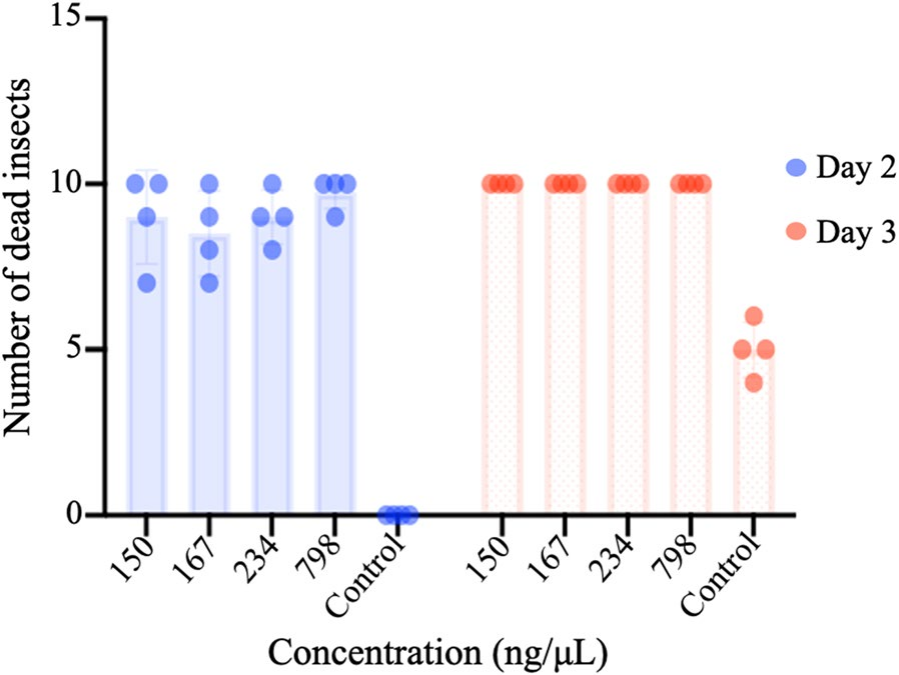
Efficacy test of purified dsRNA on *Bemisia tabaci. *The toxicity impact of different concentrations of purified dsRNA on populations (10) of *Bemisia tabaci* was evaluated by observing insect mortality on days 2 and 3 following dsRNA application. Concentrations of AREV4 dsRNA used are 150 ng/uL, 167 ng/uL, 234 ng/uL and 798 ng/uL (indicated as AREV4 -[concentration] on the chart)

The use of dsRNA for crop spray or biopesticide application is the goal of this RNAi technology. We tested the toxicity of purified bacterially derived AREV4 dsRNA on *Bemisia tabaci*. We applied dilutions of the AREV4 dsRNA (150 ng/μL, 168 ng/μL, 234 ng/μL, and 798 ng/μL) on populations (10) of *B. tabaci* in four replicated experiments using distilled water as a negative control. Analysis shows a significant difference between treated samples and negative control on day 2 (Fig. 7; Additional file 1: Tables S10–13). For the negative control, no mortality occurred on day 2, whereas about 8–10 out of 10 insects died for treated samples. No significant difference between the treatments using different dsRNA dilutions was observed either on day 2 or 3 (Additional file 1: Table S10–S13). Insects treated with dsRNA were dead, whereas approximately half of the populations were alive for negative control.

## Discussion

The increased demand for inorganic pesticide-free crop production has immensely enhanced the prospects of deploying RNAi technology to meet current agro-economic challenges (Rank & Koch, 2021). Microbial dsRNA synthesis is generally viewed as the more likely strategy to reduce the production cost in the future (Cooper et al. 2021). Although dsRNA is biosafe and eco-friendly, a crucial challenge to utilising them in agricultural applications is the cost of large-scale production (Silver et al. 2021). The direct spray of live or attenuated dsRNA-synthesising microbe on crops appears to be the most cost-effective approach. However, the engineered microbe may induce an undesirable host immune response. There is also a significant risk of environmental proliferation of the microbe (Guan et al. 2021) and the potential for interspecies transfer of plasmid-based expression elements that may lead to sustained dsRNA expression (Mendelsohn et al. 2020) that may impact non-target organisms. From this perspective, sprayable formulated “naked” bacterial-derived dsRNA is more desirable associated purification cost is a significant challenge.

The quality, yield, and cost of purified bacterially derived dsRNA significantly link to the extraction or purification method. A previous study reported that heating and sonication before dsRNA purification yielded about a 2.5–5-fold increase compared to ultrasonic crushing in conjunction with the phenol extraction (Ahn et al. 2019). A previously developed method termed RNASwift that utilises a low-cost and less hazardous reagent was shown to produce better dsRNA yield and quality than some commercial RNA extraction kits (Nwokeoji et al. 2016). Here, we scale dsRNA purification by modifying RNASwift in a strategy that does not involve toxic chemicals (e.g. phenols, chloroform) and commercial enzymes (DNases, RNases). Bacterial endogenous ribonucleases and endoribonucleases compartmentalised in the periplasmic space are known to scavenge RNA, specifically ssRNA species (Cannistraro & Kennell, 1991; Nicholson, 1999). We reasoned that lysis of the HT115 DE3 strain under non-denaturing conditions would release these endogenous ribonucleases and, therefore, could be exploited to remove ssRNA species. We modified the RNASwift method by resuspending cells in EDTA solution. EDTA induces membrane fluidisation and destabilisation (Prachayasittikul et al. 2007) and is exploited here to lyse bacteria and facilitate ribonuclease release in a process that does not denature and inactivate these enzymes. The released ribonucleases would digest ssRNA species. Additionally, other steps in the method involving chemical and pH changes degrade residual ssRNA species due to their comparatively higher chemical lability than dsRNA.

We used high-salt solutions to facilitate condensation and precipitation of the higher-molecular weight plasmids and genomic DNA on the silica matrix. We then exploited the differential solubility of DNA and RNA at low pH. Using this optimised method, we obtained a yield of approximately 88 μg per mL *E. coli* (induced in media C) culture (Fig. 3C). The results also reveal that multimeric dsRNA are usually present in dsRNA preparations (Fig. 3A). No known studies have investigated the functions of these structures in RNAi applications, but they may potentially impact the process. The dsRNA obtained from the optimised purification method is of high quality, with substantially reduced background multimeric dsRNA and no detectable DNA/ssRNA contaminants (Fig. 3B). Moreover, the obtained A260/A230 and A260/A230 ratios used to assess purified dsRNA suggests that there is no significant protein or residual salt contamination with both spectrophotometric parameters above the 1.80 thresholds (Fig. 3B).

Although large-scale fermentation for dsRNA synthesis is an established method, its viability for future agricultural applications would depend on further increasing titre rate and yield (Timmons et al. 2001) and developing cost-effective purification methods. Here, we report an approach that utilises a low-cost chemical lac-operon inducer to increase dsRNA yield. For ATU1 dsRNA, we obtained approximately 15-fold (media B), ninefold (media C), and 11-fold (media D) yield increase for formulated autoinduction media compared to 1 mM IPTG (media A) (Fig. 5A).

For AREV4 dsRNA, we see up to a sixfold increase for Media B–D (62. 49–67.79) when compared to media A (12.21) (Fig. 5B). Comparison of yields obtained from AREV4 and ATU1- dsRNA constructs does suggest that DNA template or terminator sequence may have an impact on yield. ATU1 has the same T7 RNA promoter as AREV4 but a different synthetic T7 RNA terminator. A study in 2003 utilising IPTG induction reported approximately 4 μg of dsRNA per ml of *E. coli* culture [46]. More recent studies (2013) reported 45 μg hairpin dsRNA per ml of bacteria (optical density at 600 nm = 1) (Posiri et al. 2013). Previous studies have attributed the marked difference in dsRNA yield to the fermentation methods and operation parameters (Papić et al. 2018; Thammasorn et al. 2015).

In this study, we used the batch fermentation method in flasks to compare the effect of different formulated media on productivity. Therefore, no attempt was made in our study to compare other fermentation methods or operating parameters. Consequently, we expect a proportional yield increase in more controlled and aerated fed-batch culture conditions. A previous study alleges that dsRNA titre from the fed-batch culture in a 10-L fermenter was approximately 30-fold compared to an equivalent batch fermentation (Thammasorn et al. 2015). Furthermore, optimising the nutrient media can also lead to a significant yield increase (Papić et al. 2018).

Scalability is an essential component of bioprocessing. Although most commercial RNA extraction kits are effective in lab-scale RNA extraction, the use of toxic chemicals and reagent costs makes them incompatible with industrial-scale RNA purification. Therefore, it is not only necessary to develop methods with low environmental impacts for scale RNA purification cost of reagents is a significant factor. Here, 259 mg cells induced using formulated autoinduction media (media C) were processed with the optimal dsRNA purification method (DPM2) to generate 6.39 mg dsRNA. The result demonstrates the capability of the low hazardous, cost-efficient RNA method for scaled extraction and purification of dsRNA.

Furthermore, we show in an insect toxicity test that the purified dsRNA may have a significant impact on the target insect, *Bemisia tabac*i (Fig. 6B). The limitation of this experiment is the lack of functional knockdown assay to measure the rate of gene silencing. This was beyond the scope of current study. Therefore, a future study would aim at validating the efficacy of the unformulated "naked" dsRNA and test the potency of formulated version.

## Conclusion

This work presents a high-yielding, low-cost microbial dsRNA platform with a 15-fold dsRNA yield increase compared to existing microbial dsRNA platforms. We demonstrate a substantial dsRNA yield increase compared to IPTG-based expression media using two dsRNA plasmid constructs and three formulated autoinduction media. This platform uses a dsRNA expression media that is 14 × cheaper than commercial ones. We also developed a novel, low-hazard and high-yield dsRNA purification method that uses a low-cost strategy to remove contaminating nucleic acids and cell debris. This method circumvents the need for commercial DNases, RNases, and costly, hazardous chemicals. This is in contrast with existing methods that require treatment of extracted dsRNA with RNase and DNase to remove ssRNA and DNA, respectively. We show that microbial dsRNA produces mixed populations of dsRNA, including multimeric and incomplete transcripts. We further demonstrate that our purification method eliminates these multimeric dsRNA structures. The scaled experiment yields 6.2 mg dsRNA from 259 mg wet cell pellet, demonstrating scalability and potential for application in industry-scale dsRNA purification. The production platform developed in our study will enable large-scale production of dsRNA and is suitable for industrial and low-resource settings.

### Supplementary Information

**Additional file 1: Figure S1.** **A**, **B** Analysis to establish the correlation between dsRNA yield data (AREV4 and ATU1, respectively) measured by spectrophotometric and HPLC methods. **Table S1.** Comparison of AREV4 dsRNA yield (μg per mL bacteria cell culture) purified from bacteria. **Table S2.** Comparison of AREV4 dsRNA yield (μg per mL bacteria cell culture) purified from bacteria-induced for 3- and 18 h in media A–D. **Table S3.** Descriptive statistics (Two-way ANOVA) for the effect of induction time and formulated media on AREV4 dsRNA yield. **Table S4.** Two-way ANOVA Summary Table the impact of induction time on AREV4 dsRNA yield from different formulated media. **Table S5.** Summary of total Variation (AREV4). **Table S6.** Comparison of ATU1 dsRNA yield (μg per mL bacteria cell culture) purified bacteria-induced for 3- and 18 h in media A–D. **Table S7.** Descriptive statistics (2-way ANOVA) for the effect of induction time and formulated media on ATU1 dsRNA. **Table S8.** 2-way ANOVA Summary Table for the effect of induction time on ATU1 dsRNA yield from different formulated media. **Table S9.** summary of total Variation (ATU1). **Table S10.** Impact of different concentrations of AREV4 dsRNA on Bemisia tabaci. **Table S11.** Toxicity effect of different concentrations of purified dsRNA yield on Bemisia tabaci. **Table S12.** Two-way ANOVA Summary Table for toxicity effect of different concentrations of purified dsRNA yield on Bemisia tabaci mortality rate. **Table S13.** summary of total variation (toxicity assay)

## Data Availability

All data generated, analysed and supporting this article’s conclusions are available.

## Abbreviations

ssRNA: Single-stranded ribonucleic acids
dsRNA: Double-stranded ribonucleic acids
DNase(s): Deoxyribonuclease(s)
RNase(s): Ribonuclease(s)
IPTG: Isopropyl ß-D-1-thiogalactopyranoside
IP-RP-HPLC: Ion-paired reverse-phase high-performance liquid chromatography
DPM: DsRNA purification method
