# Supplementary material for: A novel sustainable platform for scaled manufacturing of double-stranded RNA biopesticides
Source: Bioresour Bioprocess. 2022 Oct 6;9(1):107. doi: 10.1186/s40643-022-00596-2 (PMC10992233; doi:10.1186/s40643-022-00596-2)

**Supplementary Information**

**Additional File: Figure Legend**

**Figure S1** A) & B) Analysis to establish the correlation between dsRNA yield data (AREV4 and ATU1, respectively) measured by spectrophotometric and HPLC methods.

**Additional File: Table Legends:**

**Table S1** *Comparison of AREV4 dsRNA yield (*μg *per mL bacteria cell culture) purified from bacteria*

**Table S2** *Comparison of AREV4 dsRNA yield (*μg *per mL bacteria cell culture) purified from bacteria-induced for 3- and 18 hours in media A – D*

**Table S3** *Descriptive statistics (Two-way ANOVA) for the effect of induction time and formulated media on AREV4 dsRNA yield*

**Table S4** *Two-way ANOVA Summary Table the impact of induction time on AREV4 dsRNA yield from different formulated media*

**Table S5** *summary of total Variation (AREV4)*

**Table S6** *Comparison of ATU1 dsRNA yield (*μg *per mL bacteria cell culture) purified bacteria-induced for 3- and 18 hours in media A – D*

**Table S7** *Descriptive statistics (2-way ANOVA) for the effect of induction time and formulated media on ATU1 dsRNA*

**Table S8** *2-way ANOVA Summary Table for the effect of induction time on ATU1 dsRNA yield from different formulated media*

**Table S9** *summary of total Variation (ATU1)*

**Table S10** *Impact of different concentrations of AREV4 dsRNA on* *Bemisia tabaci*

**Table S11** *Toxicity effect of different concentrations of purified dsRNA yield on Bemisia tabaci*

**Table S12** *Two-way ANOVA Summary Table for toxicity effect of different concentrations of purified dsRNA yield on Bemisia tabaci mortality rate.*

**Table S13** *summary of total variation (toxicity assay)*

**Table S1** *Comparison of AREV4 dsRNA yield (*μg *per mL bacteria cell culture) purified from induced bacteria for 6- and 21 hours in media A and E*

| AREV4 | 3-hour induction | | | | 18-hour induction | | | |
| --- | --- | --- | --- | --- | --- | --- | --- | --- |
| Media A | 3.20 | 4.20 | 2.10 | 4.50 | 12.49 | 12.10 | 13.2 | 11.10 |
| Media B | 8.88 | 6.90 | 9.80 | 7.90 | 61.3 | 62.98 | 63.29 | 62.39 |
| Media C | 13.2 | 12.81 | 11.9 | 12.49 | 62.55 | 62.11 | 63.03 | 61.19 |
| Media D | 6.50 | 6.25 | 6.65 | 6.88 | 63.72 | 68.13 | 67.11 | 72.20 |

**Table S2** *Comparison of AREV4 dsRNA yield (*μg *per mL bacteria cell culture) purified from induced bacteria culture induced media A – D for 3 and 18 hours*

| Sample | AREV_A*  6-hour induction | AREV_E*  6-hour induction | AREV_A*  21-hour induction | AREV_E*  21-hour induction |
| --- | --- | --- | --- | --- |
| Replicate 1 | 10.45 | 19.2 | 5.55 | 1.02 |
| Replicate 2 | 12.33 | 18.8 | 1.93 | 1.21 |
| Replicate 3 | 11.02 | 15.8 | 2.56 | 2.66 |
| Replicate 4 | 12.21 | 19.5 | 2.01 | 3.82 |
| Replicate 5 | 11.47 | 18.8 | 3.20 | 2.52 |
| Mean = | 11.50 | 18.42 | 3.05 | 2.25 |

**Table S3** *Descriptive statistics (Two-way ANOVA) for the effect of induction time and formulated media on AREV4 dsRNA yield*

| Test details | Mean 1 | Mean 2 | Mean Diff. | SE diff. | N1 | N2 | q | DF |
| --- | --- | --- | --- | --- | --- | --- | --- | --- |
|  |  |  |  |  |  |  |  |  |
| A:3-hour vs A:18-hour | 3.500 | 12.21 | -8.710 | 1.045 | 4 | 4 | 11.79 | 24 |
| A:3-hour vs B:3-hour | 3.500 | 8.370 | -4.870 | 1.045 | 4 | 4 | 6.592 | 24 |
| A:3-hour vs B:18-hour | 3.500 | 62.49 | -58.99 | 1.045 | 4 | 4 | 79.85 | 24 |
| A:3-hour vs C:3-hour | 3.500 | 12.60 | -9.100 | 1.045 | 4 | 4 | 12.32 | 24 |
| A:3-hour vs C:18-hour | 3.500 | 62.22 | -58.72 | 1.045 | 4 | 4 | 79.48 | 24 |
| A:3-hour vs D:3-hour | 3.500 | 6.570 | -3.070 | 1.045 | 4 | 4 | 4.156 | 24 |
| A:3-hour vs D:18-hour | 3.500 | 67.79 | -64.29 | 1.045 | 4 | 4 | 87.02 | 24 |
| A:18-hour vs B:3-hour | 12.21 | 8.370 | 3.840 | 1.045 | 4 | 4 | 5.198 | 24 |
| A:18-hour vs B:18-hour | 12.21 | 62.49 | -50.28 | 1.045 | 4 | 4 | 68.06 | 24 |
| A:18-hour vs C:3-hour | 12.21 | 12.60 | -0.3900 | 1.045 | 4 | 4 | 0.5279 | 24 |
| A:18-hour vs C:18-hour | 12.21 | 62.22 | -50.01 | 1.045 | 4 | 4 | 67.69 | 24 |
| A:18-hour vs D:3-hour | 12.21 | 6.570 | 5.640 | 1.045 | 4 | 4 | 7.634 | 24 |
| A:18-hour vs D:18-hour | 12.21 | 67.79 | -55.58 | 1.045 | 4 | 4 | 75.23 | 24 |
| Test details | 8.370 | 62.49 | -54.12 | 1.045 | 4 | 4 | 73.26 | 24 |
| B:3-hour vs C:3-hour | 8.370 | 12.60 | -4.230 | 1.045 | 4 | 4 | 5.726 | 24 |
| B:3-hour vs C:18-hour | 8.370 | 62.22 | -53.85 | 1.045 | 4 | 4 | 72.89 | 24 |
| B:3-hour vs D:3-hour | 8.370 | 6.570 | 1.800 | 1.045 | 4 | 4 | 2.437 | 24 |
| B:3-hour vs D:18-hour | 8.370 | 67.79 | -59.42 | 1.045 | 4 | 4 | 80.43 | 24 |
| B:18-hour vs C:3-hour | 62.49 | 12.60 | 49.89 | 1.045 | 4 | 4 | 67.53 | 24 |
| B:18-hour vs C:18-hour | 62.49 | 62.22 | 0.2700 | 1.045 | 4 | 4 | 0.3655 | 24 |
| B:18-hour vs D:3-hour | 62.49 | 6.570 | 55.92 | 1.045 | 4 | 4 | 75.69 | 24 |
| B:18-hour vs D:18-hour | 62.49 | 67.79 | -5.300 | 1.045 | 4 | 4 | 7.174 | 24 |
| C:3-hour vs C:18-hour | 12.60 | 62.22 | -49.62 | 1.045 | 4 | 4 | 67.17 | 24 |
| C:3-hour vs D:3-hour | 12.60 | 6.570 | 6.030 | 1.045 | 4 | 4 | 8.162 | 24 |
| C:3-hour vs D:18-hour | 12.60 | 67.79 | -55.19 | 1.045 | 4 | 4 | 74.71 | 24 |
| C:18-hour vs D:3-hour | 62.22 | 6.570 | 55.65 | 1.045 | 4 | 4 | 75.33 | 24 |
| C:18-hour vs D:18-hour | 62.22 | 67.79 | -5.570 | 1.045 | 4 | 4 | 7.540 | 24 |
| D:3-hour vs D:18-hour | 6.570 | 67.79 | -61.22 | 1.045 | 4 | 4 | 82.87 | 24 |

**Table S4** *Two-way ANOVA Summary Table for the effect of induction time on AREV4 dsRNA yield from different formulated media*

| ANOVA table | SS | DF | MS | F (DFn, DFd) | P value |
| --- | --- | --- | --- | --- | --- |
| Interaction | 3349 | 3 | 1116 | F (3, 24) = 511.4 | P<0.0001 |
| Formulated media type | 5002 | 3 | 1667 | F (3, 24) = 763.7 | P<0.0001 |
| Incubation time | 15081 | 1 | 15081 | F (1, 24) = 6908 | P<0.0001 |
| Residual | 52.39 | 24 | 2.183 |  |  |

*Note*—MS = Mean squares

**Table S5** *summary of total Variation (AREV4)*

| Source of Variation | % of total variation | P value | Significant? |
| --- | --- | --- | --- |
| Interaction | 14.26 | <0.0001 | Yes |
| Formulated media type | 21.30 | <0.0001 | Yes |
| Incubation time | 64.22 | <0.0001 | Yes |

Table S6 *Comparison of ATU1 dsRNA yield (*μg *per mL bacteria cell culture) purified from bacteria culture induced in media A – D for 3 and 18 hours*

| ATU1 | 3-hour induction | | | | 18-hour induction | | | |
| --- | --- | --- | --- | --- | --- | --- | --- | --- |
| Media A | 3.55 | 4.5 | 4.25 | 3.9 | 8.21 | 9.1 | 8.31 | 7.7 |
| Media B | 18.64 | 17.54 | 18.1 | 19.28 | 123.6 | 122.55 | 116.25 | 129 |
| Media C | 47.52 | 45.21 | 42.22 | 45.25 | 77.5 | 69.3 | 75 | 76 |
| Media D | 5.75 | 8.54 | 6.96 | 6.67 | 80.5 | 89.5 | 89.2 | 95.2 |

**Table S7** *Descriptive statistics (2-way ANOVA) for the effect of induction time and formulated media on ATU1 dsRNA*

| \| Test details \| Mean 1 \| \| Mean 2 \| M.Diff. \| SE \| N1 \| \| N2 \| q \| DF \| \| --- \| --- \| --- \| --- \| --- \| --- \| --- \| --- \| --- \| --- \| --- \| \|  \| \|  \|  \|  \|  \|  \| \|  \|  \|  \| \| A:3-hour vs A:18-hour \| \| 4.05 \| 8.33 \| -4.280 \| 2.29 \| 4 \| \| 4 \| 2.64 \| 24 \| \| A:3-hour vs B:3-hour \| \| 4.05 \| 18.39 \| -14.34 \| 2.29 \| 4 \| \| 4 \| 8.85 \| 24 \| \| A:3-hour vs B:18-hour \| \| 4.05 \| 122.9 \| -118.8 \| 2.29 \| 4 \| \| 4 \| 73.28 \| 24 \| \| A:3-hour vs C:3-hour \| \| 4.05 \| 45.05 \| -41.00 \| 2.29 \| \| 4 \| 4 \| 25.29 \| 24 \| \| A:3-hour vs C:18-hour \| \| 4.05 \| 74.45 \| -70.40 \| 2.29 \| \| 4 \| 4 \| 43.43 \| 24 \| \| A:3-hour vs D:3-hour \| \| 4.05 \| 6.980 \| -2.93 \| 2.29 \| \| 4 \| 4 \| 1.807 \| 24 \| \| A:3-hour vs D:18-hour \| \| 4.05 \| 88.60 \| -84.55 \| 2.29 \| \| 4 \| 4 \| 52.15 \| 24 \| \| A:18-hour vs B:3-hour \| \| 8.33 \| 18.39 \| -10.06 \| 2.29 \| \| 4 \| 4 \| 6.205 \| 24 \| \| A:18-hour vs B:18-hour \| \| 8.33 \| 122.9 \| -114.5 \| 2.29 \| \| 4 \| 4 \| 70.64 \| 24 \| \| A:18-hour vs C:3-hour \| \| 8.33 \| 45.05 \| -36.72 \| 2.29 \| \| 4 \| 4 \| 22.65 \| 24 \| \| A:18-hour vs C:18hour \| \| 8.33 \| 74.45 \| -66.12 \| 2.29 \| \| 4 \| 4 \| 40.79 \| 24 \| \| A:18-hour vs D:3-hour \| \| 8.33 \| 6.980 \| 1.35 \| 2.29 \| \| 4 \| 4 \| 0.84 \| 24 \| \| A:18-hour vs D:18hour \| \| 8.33 \| 88.60 \| -80.27 \| 2.29 \| \| 4 \| 4 \| 49.51 \| 24 \| \| B:3-hour vs B:18hour \| \| 18.39 \| 122.9 \| -104.5 \| 2.29 \| \| 4 \| 4 \| 64.44 \| 24 \| \| B:3-hour vs C:3-hour \| \| 18.39 \| 45.05 \| -26.66 \| 2.29 \| \| 4 \| 4 \| 16.44 \| 24 \| \| B:3-hour vs C:18-hour \| \| 18.39 \| 74.45 \| -56.06 \| 2.29 \| \| 4 \| 4 \| 34.58 \| 24 \| \| B:3-hour vs D:3-hour \| \| 18.39 \| 6.980 \| 11.41 \| 2.29 \| \| 4 \| 4 \| 7.04 \| 24 \| \| B:3-hour vs D:18-hour \| \| 18.39 \| 88.60 \| -70.21 \| 2.29 \| \| 4 \| 4 \| 43.31 \| 24 \| \| B:18-hour vs C:3-hour \| \| 122.9 \| 45.05 \| 77.80 \| 2.29 \| \| 4 \| 4 \| 47.99 \| 24 \| \| B:18-hour vs C:18-hour \| \| 122.9 \| 74.45 \| 48.40 \| 2.29 \| \| 4 \| 4 \| 29.86 \| 24 \| \| B:18-hour vs D:3-hour \| \| 122.9 \| 6.980 \| 115.9 \| 2.29 \| \| 4 \| 4 \| 71.47 \| 24 \| \| B:18-hour vs D:18-hour \| \| 122.9 \| 88.60 \| 34.25 \| 2.29 \| \| 4 \| 4 \| 21.13 \| 24 \| \| C:3-hour vs C:18-hour \| \| 45.05 \| 74.45 \| -29.40 \| 2.29 \| \| 4 \| 4 \| 18.14 \| 24 \| \| C:3-hour vs D:3-hour \| \| 45.05 \| 6.980 \| 38.07 \| 2.29 \| \| 4 \| 4 \| 23.48 \| 24 \| \| C:3-hour vs D:18-hour \| \| 45.05 \| 88.60 \| -43.55 \| 2.29 \| \| 4 \| 4 \| 26.86 \| 24 \| \| C:18-hour vs D:3-hour \| \| 74.45 \| 6.980 \| 67.47 \| 2.29 \| \| 4 \| 4 \| 41.62 \| 24 \| \| C:18-hour vs D:18hour \| \| 74.45 \| 88.60 \| -14.15 \| 2.29 \| \| 4 \| 4 \| 8.73 \| 24 \| \| D:3-hour vs D:18-hour \| \| 6.980 \| 88.60 \| -81.62 \| 2.29 \| \| 4 \| 4 \| 50.35 \| 24 \| |
| --- | --- | --- | --- | --- | --- | --- | --- | --- | --- | --- | --- | --- | --- | --- | --- | --- | --- | --- | --- | --- | --- | --- | --- | --- | --- | --- | --- | --- | --- | --- | --- | --- | --- | --- | --- | --- | --- | --- | --- | --- | --- | --- | --- | --- | --- | --- | --- | --- | --- | --- | --- | --- | --- | --- | --- | --- | --- | --- | --- | --- | --- | --- | --- | --- | --- | --- | --- | --- | --- | --- | --- | --- | --- | --- | --- | --- | --- | --- | --- | --- | --- | --- | --- | --- | --- | --- | --- | --- | --- | --- | --- | --- | --- | --- | --- | --- | --- | --- | --- | --- | --- | --- | --- | --- | --- | --- | --- | --- | --- | --- | --- | --- | --- | --- | --- | --- | --- | --- | --- | --- | --- | --- | --- | --- | --- | --- | --- | --- | --- | --- | --- | --- | --- | --- | --- | --- | --- | --- | --- | --- | --- | --- | --- | --- | --- | --- | --- | --- | --- | --- | --- | --- | --- | --- | --- | --- | --- | --- | --- | --- | --- | --- | --- | --- | --- | --- | --- | --- | --- | --- | --- | --- | --- | --- | --- | --- | --- | --- | --- | --- | --- | --- | --- | --- | --- | --- | --- | --- | --- | --- | --- | --- | --- | --- | --- | --- | --- | --- | --- | --- | --- | --- | --- | --- | --- | --- | --- | --- | --- | --- | --- | --- | --- | --- | --- | --- | --- | --- | --- | --- | --- | --- | --- | --- | --- | --- | --- | --- | --- | --- | --- | --- | --- | --- | --- | --- | --- | --- | --- | --- | --- | --- | --- | --- | --- | --- | --- | --- | --- | --- | --- | --- | --- | --- | --- | --- | --- | --- | --- | --- | --- | --- | --- | --- | --- | --- | --- | --- | --- | --- | --- | --- | --- | --- | --- | --- | --- | --- | --- | --- | --- | --- | --- | --- | --- | --- | --- | --- | --- | --- | --- | --- | --- | --- | --- | --- | --- | --- | --- | --- | --- | --- | --- | --- | --- | --- | --- | --- | --- | --- | --- | --- | --- | --- | --- | --- | --- | --- | --- | --- | --- | --- | --- | --- | --- | --- | --- | --- | --- | --- |

Note: A, B, C and D denoting media A – D; 3-hour and 18-hour, respectively representing 3- and 18-hour induction. M. Diff = mean difference.

**Table S8** *Two-way ANOVA Summary Table for the effect of induction time on ATU1 dsRNA yield from different formulated media*

| ANOVA table | SS | DF | MS | F (DFn, DFd) | P value |
| --- | --- | --- | --- | --- | --- |
| Interaction | 12766 | 3- | 4255 | F (3-, 24) = 404.8 | P<0.0001 |
| Formulated media | 19066 | 3- | 63-55 | F (3-, 24) = 604.5 | P<0.0001 |
| Induction time | 24147 | 1 | 24147 | F (1, 24) = 2297 | P<0.0001 |
| Residual | 252.3- | 24 | 10.51 |  |  |

**Table S9** *summary of total Variation (ATU1)*

| Source of Variation | % of total variation | P value | Significant? |
| --- | --- | --- | --- |
| Interaction | 22.70 | <0.0001 | Yes |
| Expression media formulation | 33.91 | <0.0001 | Yes |
| Induction time | 42.94 | <0.0001 | Yes |

**Table S10** *Impact of different concentrations of AREV4 dsRNA on* *Bemisia tabaci*

| AREV4 sRNA conc. (ng/uL) | Day 2 (Number of insect dead) | | | | Day 3 (Number of insect dead) | | | | |  |
| --- | --- | --- | --- | --- | --- | --- | --- | --- | --- | --- |
| 150 | 9 | 10 | 7 | 10 | | 10 | 10 | 10 | 10 | |
| 167 | 8 | 7 | 10 | 9 | | 10 | 10 | 10 | 10 | |
| 234 | 8 | 10 | 9 | 9 | | 10 | 10 | 10 | 10 | |
| 798 | 10 | 10 | 9 | 10 | | 10 | 10 | 10 | 10 | |
| Control | 0 | 0 | 0 | 0 | | 5 | 4 | 6 | 5 | |

**Table S11** *Descriptive statistics (Two-way ANOVA) for Toxicity effect of different concentrations of purified dsRNA yield on Bemisia tabaci*

| **Test details** | **Mean 1** | | **Mean**  **2** | **Mean Diff.** | **SE of diff.** | **N1** | **N2** | **q** | **DF** |
| --- | --- | --- | --- | --- | --- | --- | --- | --- | --- |
|  |  |  | |  |  |  |  |  |  |
| **150: DAY 2 vs 150: DAY 3** | 9.000 | 10.00 | | -1.000 | 0.5123 | 4 | 4 | 2.760 | **30** |
| **150: DAY 2 vs 167: DAY 2** | 9.000 | 8.500 | | 0.5000 | 0.5123 | 4 | 4 | 1.380 | **30** |
| **150: DAY 2 vs 167: DAY 3** | 9.000 | 10.00 | | -1.000 | 0.5123 | 4 | 4 | 2.760 | **30** |
| **150: DAY 2 vs 234: DAY 2** | 9.000 | 9.000 | | 0.000 | 0.5123 | 4 | 4 | 0.000 | **30** |
| **150: DAY 2 vs 234: DAY 3** | 9.000 | 10.00 | | -1.000 | 0.5123 | 4 | 4 | 2.760 | **30** |
| **150: DAY 2 vs 798: DAY 2** | 9.000 | 9.750 | | -0.7500 | 0.5123 | 4 | 4 | 2.070 | **30** |
| **150: DAY 2 vs 798: DAY 3** | 9.000 | 10.00 | | -1.000 | 0.5123 | 4 | 4 | 2.760 | **30** |
| **150: DAY 2 vs C: DAY 2** | 9.000 | 0.000 | | 9.000 | 0.5123 | 4 | 4 | 24.84 | **30** |
| **150: DAY 2 vs C: DAY 3** | 9.000 | 5.000 | | 4.000 | 0.5123 | 4 | 4 | 11.04 | **30** |
| **150: DAY 3 vs 167: DAY 2** | 10.00 | 8.500 | | 1.500 | 0.5123 | 4 | 4 | 4.140 | **30** |
| **150: DAY 3 vs 167: DAY 3** | 10.00 | 10.00 | | 0.000 | 0.5123 | 4 | 4 | 0.000 | **30** |
| **150: DAY 3 vs 234: DAY 2** | 10.00 | 9.000 | | 1.000 | 0.5123 | 4 | 4 | 2.760 | **30** |
| **150: DAY 3 vs 234: DAY 3** | 10.00 | 10.00 | | 0.000 | 0.5123 | 4 | 4 | 0.000 | **30** |
| **150: DAY 3 vs 798: DAY 2** | 10.00 | 9.750 | | 0.2500 | 0.5123 | 4 | 4 | 0.6901 | **30** |
| **150: DAY 3 vs 798: DAY 3** | 10.00 | 10.00 | | 0.000 | 0.5123 | 4 | 4 | 0.000 | **30** |
| **150: DAY 3 vs C: DAY 2** | 10.00 | 0.000 | | 10.00 | 0.5123 | 4 | 4 | 27.60 | **30** |
| **150: DAY 3 vs C: DAY 3** | 10.00 | 5.000 | | 5.000 | 0.5123 | 4 | 4 | 13.80 | **30** |
| **167: DAY 2 vs 167: DAY 3** | 8.500 | 10.00 | | -1.500 | 0.5123 | 4 | 4 | 4.140 | **30** |
| **167: DAY 2 vs 234: DAY 2** | 8.500 | 9.000 | | -0.5000 | 0.5123 | 4 | 4 | 1.380 | **30** |
| **167: DAY 2 vs 234: DAY 3** | 8.500 | 10.00 | | -1.500 | 0.5123 | 4 | 4 | 4.140 | **30** |
| **167: DAY 2 vs 798: DAY 2** | 8.500 | 9.750 | | -1.250 | 0.5123 | 4 | 4 | 3.450 | **30** |
| **167: DAY 2 vs 798: DAY 3** | 8.500 | 10.00 | | -1.500 | 0.5123 | 4 | 4 | 4.140 | **30** |
| **167: DAY 2 vs C: DAY 2** | 8.500 | 0.000 | | 8.500 | 0.5123 | 4 | 4 | 23.46 | **30** |
| **167: DAY 2 vs C: DAY 3** | 8.500 | 5.000 | | 3.500 | 0.5123 | 4 | 4 | 9.661 | **30** |
| **167: DAY 3 vs 234: DAY 2** | 10.00 | 9.000 | | 1.000 | 0.5123 | 4 | 4 | 2.760 | **30** |
| **167: DAY 3 vs 234: DAY 3** | 10.00 | 10.00 | | 0.000 | 0.5123 | 4 | 4 | 0.000 | **30** |
| **167: DAY 3 vs 798: DAY 2** | 10.00 | 9.750 | | 0.2500 | 0.5123 | 4 | 4 | 0.6901 | **30** |
| **167: DAY 3 vs 798: DAY 3** | 10.00 | 10.00 | | 0.000 | 0.5123 | 4 | 4 | 0.000 | **30** |
| **167: DAY 3 vs C: DAY 2** | 10.00 | 0.000 | | 10.00 | 0.5123 | 4 | 4 | 27.60 | **30** |
| **167: DAY 3 vs C: DAY 3** | 10.00 | 5.000 | | 5.000 | 0.5123 | 4 | 4 | 13.80 | **30** |
| **234: DAY 2 vs 234: DAY 3** | 9.000 | 10.00 | | -1.000 | 0.5123 | 4 | 4 | 2.760 | **30** |
| **234: DAY 2 vs 798: DAY 2** | 9.000 | 9.750 | | -0.7500 | 0.5123 | 4 | 4 | 2.070 | **30** |
| **234: DAY 2 vs 798: DAY 3** | 9.000 | 10.00 | | -1.000 | 0.5123 | 4 | 4 | 2.760 | **30** |
| **234: DAY 2 vs C: DAY 2** | 9.000 | 0.000 | | 9.000 | 0.5123 | 4 | 4 | 24.84 | **30** |
| **234: DAY 2 vs C: DAY 3** | 9.000 | 5.000 | | 4.000 | 0.5123 | 4 | 4 | 11.04 | **30** |
| **234: DAY 3 vs 798: DAY 2** | 10.00 | 9.750 | | 0.2500 | 0.5123 | 4 | 4 | 0.6901 | **30** |
| **234: DAY 3 vs 798: DAY 3** | 10.00 | 10.00 | | 0.000 | 0.5123 | 4 | 4 | 0.000 | **30** |
| **234: DAY 3 vs C: DAY 2** | 10.00 | 0.000 | | 10.00 | 0.5123 | 4 | 4 | 27.60 | **30** |
| **234: DAY 3 vs C: DAY 3** | 10.00 | 5.000 | | 5.000 | 0.5123 | 4 | 4 | 13.80 | **30** |
| **798: DAY 2 vs 798: DAY 3** | 9.750 | 10.00 | | -0.2500 | 0.5123 | 4 | 4 | 0.6901 | **30** |
| **798: DAY 2 vs C: DAY 2** | 9.750 | 0.000 | | 9.750 | 0.5123 | 4 | 4 | 26.91 | **30** |
| **798: DAY 2 vs C: DAY 3** | 9.750 | 5.000 | | 4.750 | 0.5123 | 4 | 4 | 13.11 | **30** |
| **798: DAY 3 vs C: DAY 2** | 10.00 | 0.000 | | 10.00 | 0.5123 | 4 | 4 | 27.60 | **30** |
| **798: DAY 3 vs C: DAY 3** | 10.00 | 5.000 | | 5.000 | 0.5123 | 4 | 4 | 13.80 | **30** |
| **C: DAY 2 vs C: DAY 3** | **0.000** | **5.000** | | **-5.000** | **0.5123** | **4** | **4** | **13.80** | **30** |

Note: C denotes Control.

**Table S12** *Two-way ANOVA Summary Table for toxicity effect of different concentrations of purified dsRNA yield on Bemisia tabaci mortality rate*

| ANOVA table | SS | DF | MS | F (DFn, DFd) | P value |
| --- | --- | --- | --- | --- | --- |
| Interaction | 28.00 | 4 | 7.000 | F (4, 30) = 13.33 | P<0.0001 |
| dsRNA treatment | 318.0 | 4 | 79.50 | F (4, 30) = 151.4 | P<0.0001 |
| Death rate (days) | 30.63 | 1 | 30.63 | F (1, 30) = 58.33 | P<0.0001 |
| Residual | 15.75 | 30 | 0.5250 |  |  |

**Table S13** *summary of total variation*

| Source of Variation | % of total variation | P value | Significant? |
| --- | --- | --- | --- |
| Interaction | 7.136 | <0.0001 | Yes |
| dsRNA treatment | 81.04 | <0.0001 | Yes |
| Death rate (days) | 7.805 | <0.0001 | Yes |


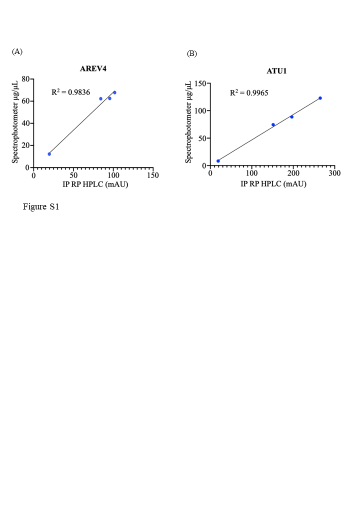

Supplement: Supplementary file 1 — Additional file 1: Figure S1. A, B Analysis to establish the correlation between dsRNA yield data (AREV4 and ATU1, respectively) measured by spectrophotometric and HPLC methods. Table S1. Comparison of AREV4 dsRNA yield (μg per mL bacteria cell culture) purified from bacteria. Table S2. Comparison of AREV4 dsRNA yield (μg per mL bacteria cell culture) purified from bacteria-induced for 3- and 18 h in media A–D. Table S3. Descriptive statistics (Two-way ANOVA) for the effect of induction time and formulated media on AREV4 dsRNA yield. Table S4. Two-way ANOVA Summary Table the impact of induction time on AREV4 dsRNA yield from different formulated media. Table S5. Summary of total Variation (AREV4). Table S6. Comparison of ATU1 dsRNA yield (μg per mL bacteria cell culture) purified bacteria-induced for 3- and 18 h in media A–D. Table S7. Descriptive statistics (2-way ANOVA) for the effect of induction time and formulated media on ATU1 dsRNA. Table S8. 2-way ANOVA Summary Table for the effect of induction time on ATU1 dsRNA yield from different formulated media. Table S9. summary of total Variation (ATU1). Table S10. Impact of different concentrations of AREV4 dsRNA on Bemisia tabaci. Table S11. Toxicity effect of different concentrations of purified dsRNA yield on Bemisia tabaci. Table S12. Two-way ANOVA Summary Table for toxicity effect of different concentrations of purified dsRNA yield on Bemisia tabaci mortality rate. Table S13. summary of total variation (toxicity assay) [file 40643_2022_596_MOESM1_ESM.docx]
